# Reducing echocardiographic examination time through routine use of fully automated software: a comparative study of measurement and report creation time

**DOI:** 10.1007/s12574-023-00636-6

**Published:** 2024-02-03

**Authors:** Yukina Hirata, Yuka Nomura, Yoshihito Saijo, Masataka Sata, Kenya Kusunose

**Affiliations:** 1 Ultrasound Examination Center, Tokushima University Hospital, Tokushima, Japan; 2 Department of Cardiovascular Medicine, Tokushima University Hospital, Tokushima, Japan; 3 Department of Cardiovascular Medicine, Nephrology, and Neurology, Graduate School of Medicine, University of the Ryukyus, 207 Uehara, Nishihara Town, Okinawa, Japan

**Keywords:** Echocardiography, Artificial intelligence, Deep learning

## Abstract

**Background:**

Manual interpretation of echocardiographic data is time-consuming and operator-dependent. With the advent of artificial intelligence (AI), there is a growing interest in its potential to streamline echocardiographic interpretation and reduce variability. This study aimed to compare the time taken for measurements by AI to that by human experts after converting the acquired dynamic images into DICOM data.

**Methods:**

Twenty-three consecutive patients were examined by a single operator, with varying image quality and different medical conditions. Echocardiographic parameters were independently evaluated by human expert using the manual method and the fully automated US2.ai software. The automated processes facilitated by the US2.ai software encompass real-time processing of 2D and Doppler data, measurement of clinically important variables (such as LV function and geometry), automated parameter assessment, and report generation with findings and comments aligned with guidelines. We assessed the duration required for echocardiographic measurements and report creation.

**Results:**

The AI significantly reduced the measurement time compared to the manual method (159 ± 66 vs. 325 ± 94 s, *p* < 0.01). In the report creation step, AI was also significantly faster compared to the manual method (71 ± 39 vs. 429 ± 128 s, *p* < 0.01). The incorporation of AI into echocardiographic analysis led to a 70% reduction in measurement and report creation time compared to manual methods. In cases with fair or poor image quality, AI required more corrections and extended measurement time than in cases of good image quality. Report creation time was longer in cases with increased report complexity due to human confirmation of AI-generated findings.

**Conclusions:**

This fully automated software has the potential to serve as an efficient tool for echocardiographic analysis, offering results that enhance clinical workflow by providing rapid, zero-click reports, thereby adding significant value.

**Supplementary Information:**

The online version contains supplementary material available at 10.1007/s12574-023-00636-6.

## Introduction

Echocardiography, a widely used imaging modality for assessing cardiac structure and function, involves the acquisition of images and subsequent measurement of various parameters [1]. However, the traditional interpretation of echocardiographic images requires manual analysis by trained experts, leading to time-consuming and operator-dependent results [2, 3]. The use of artificial intelligence (AI) in medical imaging has attracted attention due to its potential to improve examination efficiency, consistency, and accuracy over human interpretation [4, 5]. Several studies have demonstrated the potential of deep learning algorithms in classifying echocardiographic images based on specific view classifications, quantification of cardiac volumes, and assessment of cardiac systolic function [6–10]. In previous studies, several groups developed and externally validated an automated deep learning-based workflow for the classification and annotation of echocardiographic images [11, 12].

Therefore, there is a need to explore the use of AI algorithms to automate the measurement process and potentially reduce the overall examination time. The hypothesis of this study is that the implementation of AI algorithms for echocardiographic parameter measurement, after converting dynamic images to DICOM data, will lead to a significant reduction in measurement time compared to manual measurements performed by human experts. The AI's ability to automatically recognize and measure various cardiac parameters is expected to expedite the analysis process and provide efficient and reliable results, potentially revolutionizing the field of echocardiography [13]. We designed a prospective, single center, pilot study aimed to compare the time required for the measurement and report creation using conventional manual methods vs fully automated DICOM reading software (US2.ai).

## Methods

### Study population

**Fig. 1:**
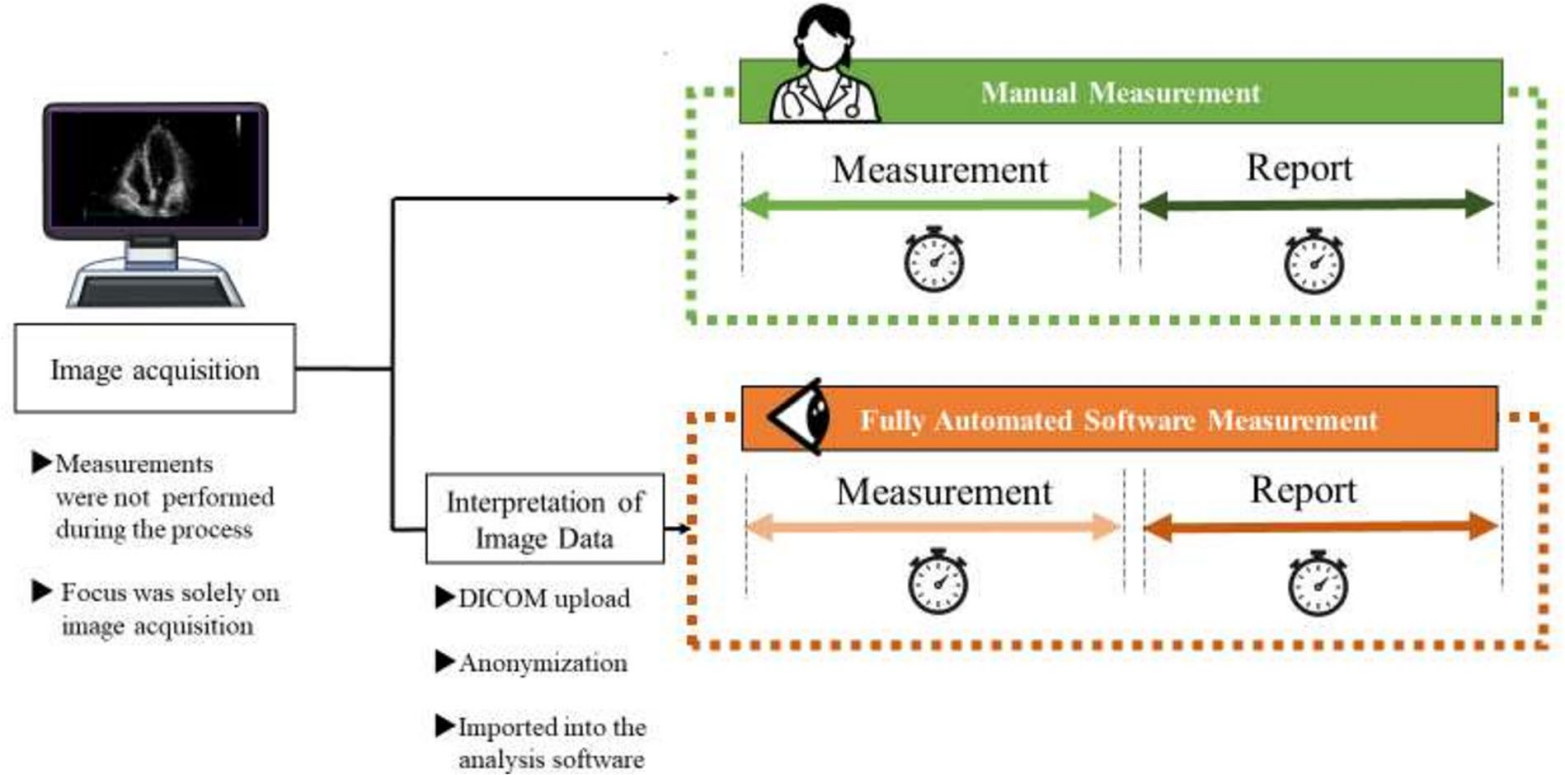
Flow chart of echocardiographic parameter measurement. The study population and echocardiographic parameter measurement. The same 2D videos and images were used for measurements by both human examiners and AI

The study enrolled patients who underwent echocardiographic evaluation conducted by a specific sonographer. Patients diagnosed with arrhythmia or poor image quality were also included in this study. Figure 1 shows the study workflow for echocardiographic parameter measurements. In all cases of echocardiographic examination, measurements were not performed during the recording process, with the focus solely on image acquisition. Subsequently, the time required for measurement and the creation of echocardiographic reports was recorded for both the experienced human examiner using the manual method and the fully automated analysis software. The start point for the manual measurement was defined as "The initial measurement image appears" and the endpoint was "Completion of all measurements". For the report creation step, the start point for the manual report creation was defined as "Entering initial measurement values" and the endpoint was "Completion of report comments entry". For the fully automated software, report creation initiated upon "Patient screen appears" and concluded with "Confirmation and modification fully completed." The fully automated software process was conducted by the same examiner, approximately one month after the measurements were taken using the manual method.

### Echocardiographic image acquisition

Echocardiography was performed using commercially available ultrasound machines. Image acquisition was performed by an experienced technician who holds certification as an echocardiography technologist recognized by the Japanese Society of Echocardiography. Only the necessary images for these parameters were recorded, and no measurements were performed during the examination. The image quality was categorized into three grades for subsequent assessment by the same observer. The quality of left ventricle (LV) images was assessed by considering the visibility of segments and the extent of endocardial border delineation in three cardiac apex sections. The evaluation criteria were as follows: good (0–2 segments poorly visible), fair (3–5 segments poorly visible), and poor (> 5 segments poorly visible). Similarly, Doppler image quality was also evaluated using a similar three-grade system. This evaluation specifically focused on the clarity of Doppler envelopes, with classifications of good (clear envelopes), fair (partially clear envelopes), and poor (unclear envelopes).

### Manual assessment

All parameters were selected and measured following the routine examination protocols at our facility in accordance with the guidelines recommended by the American Society of Echocardiography [14]. Apical two- and four-chamber images were included. The biplane method of disks in two dimensions was used to calculate the volumes of the LV. The LV ejection fraction (LVEF) was determined using these volumes. Left atrial (LA) volume was also calculated using the biplane method of disks in two dimensions. Echocardiographic images were obtained for measurements of various parameters, including the interventricular septal thickness in diastole (IVSd), left ventricular internal diameter in diastole and systole (LVIDd, LVIDs), left ventricular posterior wall thickness in diastole (LVPWd), left ventricular mass index (LVMi), relative wall thickness (RWT), the left ventricular end diastolic and systolic volume by the modified Simpson's biplane method (LVEDV and LVESV MOD biplane), and the left ventricular ejection fraction by the modified Simpson's biplane method (LVEF MOD biplane). Images were included for Doppler parameter measurements such as the left and right ventricular outflow tract peak velocities (LVOT Vmax, RVOT Vmax), the aortic valve peak velocity (AoV Vmax), the mitral valve E and A wave velocities (MV-E, MV-A), the deceleration time (DecT), the early and late diastolic tissue Doppler velocities at the lateral and septal mitral annulus (eʹ lateral, eʹ septal, aʹ lateral, aʹ septal), the systolic tissue Doppler velocities at the lateral and septal mitral annulus (sʹ lateral, sʹ septal), the tricuspid regurgitant peak velocity (TR Vmax), the tricuspid annular plane systolic excursion (TAPSE), and the systolic, early diastolic and late diastolic tissue Doppler velocity at the tricuspid annulus (sʹ TAM, eʹ TAM, aʹ TAM). In the manual method, the findings from echocardiographic examinations, including measured values, were documented in text format to ensure clear understanding in a clinical context. These reports contained information about the presence of left ventricular and left atrial enlargement, right ventricular and right atrial enlargement, left ventricular wall hypertrophy, and wall motion abnormalities. Furthermore, the reports covered aspects, such as diastolic function, valvular diseases, and pulmonary hypertension.

### Fully automated software

The study employed the US2.ai software, a fully automated DICOM reading software known for its speed and compatibility with various echo devices [15]. This software processed the 2D, Doppler in real-time, zero-click complete reports. The variables are measurements deemed clinically important by international societies (European Association of Cardiovascular Imaging [EACVI] [16], American Society of Echocardiography [ASE] [14]) for a comprehensive transthoracic adult echocardiogram. In this software, measurements equivalent to expert readings were attainable. However, manual adjustments were made in the following cases: (1) when measurements were missing despite the presence of images, and (2) when inaccuracies in measurements or misidentification of images were identified. The time required for these corrections was included in the measurement process.

In the report creation process, LV systolic function, LV diastolic function, LV geometry, RV function, LV and RV size, LA and RA size, the presence of aortic stenosis, pulmonary hypertension, as well as clinical considerations were automatically evaluated. All findings were accompanied by comments following multiple guidelines based on the acquired values. Any missing comments, such as asynergy or valve regurgitation, were manually added by a human in this study. In our study, we defined 'negative report complexity' as cases where the patient's cardiac function is appropriate for their age and does not show any significant abnormalities. Conversely, 'positive report complexity' was assigned to scenarios involving more complicated clinical conditions. This encompasses patients with reduced EF, heart failure, valvular heart disease, pulmonary hypertension, or a combination of these issues. Moreover, cases presenting findings not automatically detected by the software, such as significant valve regurgitation, were also categorized under positive report complexity. Once a report containing clinical results in a format comparable (or not inferior) to those obtained through the manual method was generated, the report creation process was deemed complete.

### Statistical analysis

Continuous data were expressed as mean ± standard deviations (SD) and categorical data as an absolute number and percentages. Student’s *t*-test was used to compare continuous variables while the Chi-square test was used to compare categorical variables. Agreements between expert human and fully automated measurements for continuous variables were assessed using Intraclass Correlation Coefficients (ICC). Statistical analyses were performed using SPSS 21.0 (SPSS, Chicago, IL, USA) and MedCalc 19.5.6 (Mariakerke, Belgium). *P* value < 0.05 was considered statistically significant.

### Sample size determination

We performed sample size calculations using the following methodology. The total time for the manual process was estimated at 23 min based on input from multiple individuals. Furthermore, drawing insights from various sources, we projected an examination time of 15 min when utilizing AI, indicating an expected time difference of 8 min compared to the manual method. The SD for the manual process was assumed to be 10 min. Additionally, we hypothesized that the AI method would consistently yield time savings compared to the manual approach. The objective was to determine the minimum required sample size to detect this difference, considering an 80% statistical power and a significance level of 5%. Employing a paired t-test model, we utilized the mean difference and SD between manual and AI measurements for our calculations. Based on our analyses, we concluded that a sample size of approximately 21 participants per group would yield statistically significant results when the AI method is employed and the SD is approximately 15.

## Results

### Clinical backgrounds

We investigated a cohort of 23 subjects, which consisted of the required minimum sample size of 21 cases, along with an additional preliminary inclusion of 2 cases (mean age; 57 ± 17 years, 30% males). Patient details are provided in Supplementary Table 1 and Supplementary Table 2. The distribution of echocardiogram requests in the study cohort was as follows: 13 cases were designated for screening, 4 cases pertained to diagnosed and monitored ischemic heart disease, and 2 cases sought an assessment for arrhythmia. Furthermore, individual cases of hypertrophic cardiomyopathy, severe pulmonary hypertension and severe aortic stenosis were observed. Additionally, one case required an echocardiogram to be conducted in the intensive care unit. The echocardiographic image quality was categorized as follows: 16 cases were rated as good, 6 cases as fair, and 1 case as poor.

### Measurement and report creation by AI

Table 1 indicates the count of measurements successfully captured by AI across various echocardiographic parameters. The following parameters were recognized and measured by the AI with a success rate of 100%: LVIDd, LVIDs, IVSd, LVPWd, LV mass, RWT, LVEDV MOD biplane, LVESV MOD biplane, LVEF MOD biplane, LAESV MOD biplane, MV-E, Dec T, E/eʹ lateral, sʹ lateral, eʹ lateral, TR V max, LVOTd, LVOT Vmax, RVOT Vmax, and AoV Vmax. However, AI was unable to evaluate E/A, MV-A, aʹ lateral, sʹ TAM, and eʹ TAM in one case. Additionally, the E/eʹ mean, eʹ lateral, eʹ septal, aʹ septal, and aʹ TAM measurements were not recognized by AI, resulting in inaccuracies in two cases. For all parameters that significantly deviated from expert measurements, adjustments were made. Analysis using ICC indicated a high level of agreement, with *p* values < 0.05, between expert human and fully automated measurements for all these parameters.

**Table 1:** The comparison of echocardiographic parameters and time required between manual and the AI

	Manual		AI		ICC (95%CI)	*p* value
	*n*	Mean ± SD	*n*	Mean ± SD		
Echocardiographic parameters						
IVSd, mm	23	8.0 ± 2.2	23	8.6 ± 2.2	0.84 (0.67–0.93)	** < 0.01**
LVIDd, mm	23	47 ± 6	23	45 ± 6	0.81 (0.60–0.91)	** < 0.01**
LVIDs, mm	23	30 ± 7	23	29 ± 6	0.81 (0.61–0.92)	** < 0.01**
LVPWd, mm	23	7.8 ± 1.3	23	8.2 ± 1.2	0.63 (0.31–0.82)	** < 0.01**
LVMI, g/m^2^	23	80 ± 30	23	85 ± 34	0.69 (0.40–0.86)	** < 0.01**
RWT	23	0.3 ± 0.1	23	0.4 ± 0.1	0.52 (0.16–0.76)	** < 0.01**
LVEDV MOD biplane, ml	23	96 ± 40	23	77 ± 37	0.84 (0.65–0.93)	** < 0.01**
LVESV MOD biplane, ml	23	42 ± 30	23	35 ± 32	0.96 (0.90–0.95)	** < 0.01**
LVEF MOD biplane, %	23	62 ± 10	23	62 ± 11	0.88 (0.74–0.95)	** < 0.01**
LAESV MOD biplane, ml	22	43 ± 22	22	41 ± 20	0.96 (0.90–0.98)	** < 0.01**
LVOTd, mm	23	20 ± 3	23	19 ± 2	0.91 (0.79–0.96)	** < 0.01**
LVOT Vmax, cm/s	23	97 ± 21	23	93 ± 20	0.91 (0.80–0.96)	** < 0.01**
RVOT Vmax, cm/s	21	70 ± 16	21	58 ± 15	0.90 (0.76–0.96)	** < 0.01**
AoV Vmax, m/s	23	1.6 ± 0.7	23	1.6 ± 0.7	1.00 (0.99–1.00)	** < 0.01**
E/A	22	1.2 ± 0.4	21	1.2 ± 0.4	0.93 (0.85–0.97)	** < 0.01**
MV-E, cm/s	23	72 ± 19	23	74 ± 20	0.99 (0.97–0.99)	** < 0.01**
MV-A, cm/s	22	65 ± 15	22	66 ± 15	0.96 (0.91–0.98)	** < 0.01**
DecT, ms	23	208 ± 48	23	212 ± 46	0.48 (0.09–0.75)	**0.01**
E/eʹ mean	22	10.0 ± 5.0	20	9.6 ± 5.6	0.85 (0.66–0.94)	** < 0.01**
sʹ lateral, cm/s	22	7.8 ± 2.7	22	8.8 ± 2.6	0.63 (0.30–0.83)	** < 0.01**
sʹ septal, cm/s	22	7.0 ± 2.1	20	7.6 ± 2.5	0.89 (0.73–0.96)	** < 0.01**
eʹ lateral, cm/s	22	9.1 ± 4.4	22	9.9 ± 4.4	0.93 (0.84–0.97)	** < 0.01**
eʹ septal, cm/s	22	7.3 ± 3.2	20	7.4 ± 3.3	0.93 (0.81–0.97)	** < 0.01**
aʹ lateral, cm/s	22	9.0 ± 2.9	21	9.1 ± 3.0	0.89 (0.76–0.95)	** < 0.01**
aʹ septal, cm/s	21	8.2 ± 2.3	19	8.2 ± 2.6	0.89 (0.71–0.96)	** < 0.01**
TR Vmax, m/s	23	2.4 ± 0.5	23	2.5 ± 0.5	0.94 (0.86–0.97)	** < 0.01**
TAPSE, mm	22	20 ± 4.3	22	21 ± 4.7	0.77 (0.47–0.90)	** < 0.01**
sʹ TAM, cm/s	22	11.3 ± 3.0	21	12.3 ± 3.0	0.81 (0.60–0.92)	** < 0.01**
eʹ TAM, cm/s	22	10.8 ± 5.0	21	12.0 ± 4.8	0.94 (0.87–0.98)	** < 0.01**
aʹ TAM, cm/s	21	12.2 ± 3.5	19	12.5 ± 3.6	0.94 (0.86–0.98)	** < 0.01**
Time						
Measurement, s	325 ± 94		159 ± 66			** < 0.01**
Report creation, s	429 ± 128		71 ± 39			** < 0.01**
Measurement + Report, s	754 ± 206		230 ± 83			** < 0.01**

Data are presented as number of patients or mean ± SD

IVSd, interventricular septal thickness in diastole; LVIDd, left ventricular internal diameter in diastole; LVIDs, left ventricular internal diameter in systole; LVPWd, left ventricular posterior wall thickness in diastole; LVMi, left ventricular mass index, RWT, relative wall thickness; LVEDV MOD biplane, left ventricular end diastolic volume by the modified Simpson's biplane method; LVESV MOD biplane, left ventricular end systolic volume by the modified Simpson's biplane method; LVEF MOD biplane, left ventricular ejection fraction by the modified Simpson's biplane method; LVOT Vmax, left ventricular outflow tract peak velocity; RVOT Vmax, right ventricular outflow tract peak velocity; AoV Vmax, aortic valve peak velocity; MV-E, mitral valve E wave velocity; MV-A, mitral valve A wave velocity; DecT, deceleration time; eʹ lateral, early diastolic tissue Doppler velocity at the lateral mitral annulus; eʹ septal, early diastolic tissue Doppler velocity at the septal mitral annulus; aʹ lateral, late diastolic tissue Doppler velocity at the lateral mitral annulus lateral; aʹ septal, late diastolic tissue Doppler velocity at the septal mitral annulus; sʹ lateral, systolic tissue Doppler velocity at the lateral mitral annulus; sʹ septal, systolic tissue Doppler velocity at the septal mitral annulus; TR Vmax, tricuspid regurgitant peak velocity; TAPSE, tricuspid annular plane systolic excursion; Sʹ TAM, systolic tissue Doppler velocity at the tricuspid annulus

### Time required for AI and manual methods

**Fig. 2:**
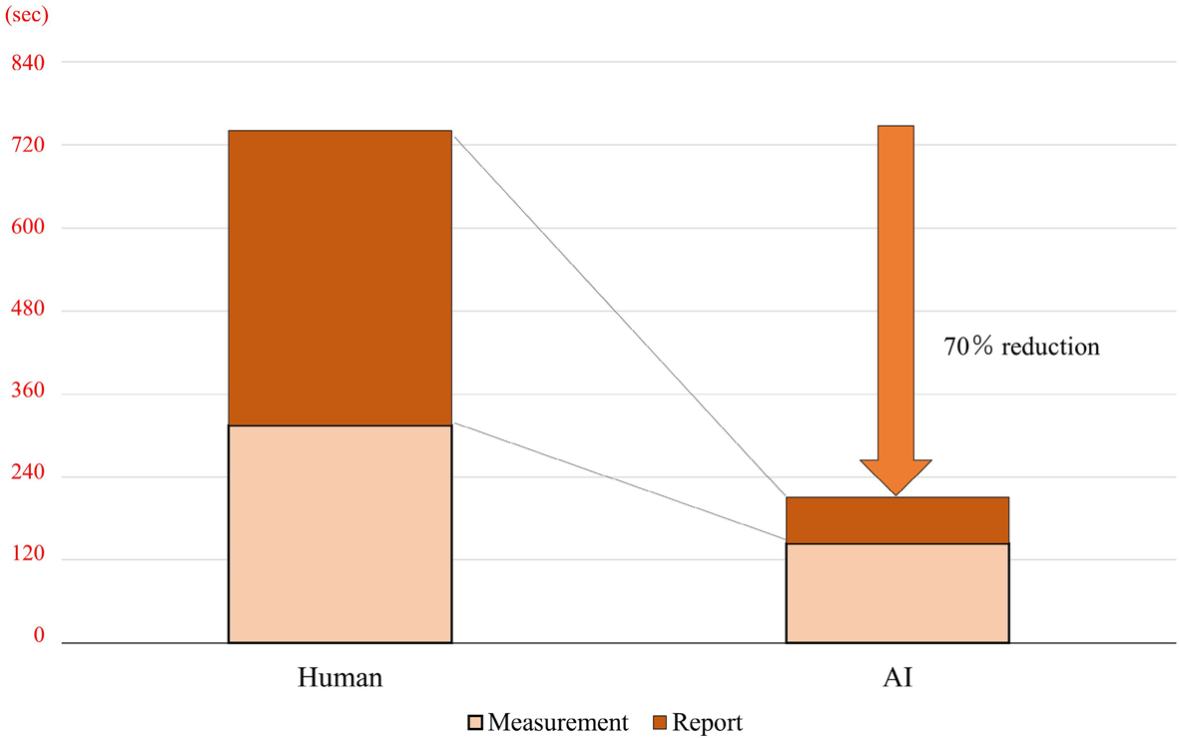
Time difference in echocardiographic measurement and report creation between Human and AI. Compared to the time required for measurements and report creation by humans, using AI enabled an average reduction of 70% in time

As shown in Supplementary Table 1 and Supplementary Table 2, AI achieved time savings of 96% for measurements and 100% for report creation compared to the manual method. Table 1 presents a comparison of measurement time and report creation times between the manual and AI methods. The manual method required an average measurement time of 325 ± 94 s, while AI took 159 ± 66 s (*p* < 0.01). In the report creation step, the average time for manual report creation was 429 ± 128 s, whereas AI only needed 71 ± 39 s (*p* < 0.01). Overall, AI significantly reduced the time required for measurement and report creation compared to the manual method (230 ± 83 vs 754 ± 206 s, *p* < 0.01). As depicted in Fig. 2, the average time for measurement and report creation per case can be reduced by 524 s (70%) due to the significant time reduction achieved by AI compared to the manual method.

### Impact of AI on measurement and report creation time

The median AI measurement time was 217 s, leading to the division of patients into two groups based on this median value. Table 2 shows the characteristics of the two groups based on AI time. The group with faster measurements showed significantly fewer modified indications compared to the group with longer measurements (2.3 ± 1.9 vs. 5.2 ± 2.6, *p* < 0.01). The faster measurement group showed a lower percentage of patients with fair or poor image quality (9% vs. 50%, *p* = 0.02) and more than mild pericardial effusion (0% vs 33%, *p* = 0.02) compared to the longer measurement group. Additionally, the number of diagnoses in patients' reports was lower in the faster measurement group (0.7 ± 0.9 vs 2.8 ± 2.1, *p* < 0.01). Even when Doppler image envelopes were classified as 'fair', they usually matched expert measurements, rarely requiring further adjustments or re-measurements (9% vs. 17%, *p* = 0.30).

**Table 2:** Characteristics of groups with and without time requirements

	All *n* = 23	< 217 s *n* = 11	≥ 217 s *n* = 12	*p* value
Age, years	57 ± 17	54 ± 17	60 ± 17	0.23
Male, *n* (%)	7 (30)	1 (9)	6 (50)	**0.02**
Height, m	1.59 ± 1.0	1.57 ± 0.8	1.63 ± 1.3	0.12
Weight, kg	58 ± 12	57 ± 12	60 ± 12	0.27
Body surface area	1.60 ± 0.19	1.55 ± 0.18	1.64 ± 0.19	0.16
Heart rate, bpm	78 ± 17	83 ± 17	73 ± 15	0.07
Systolic blood pressure, mmHg	117 ± 20	121 ± 20	114 ± 21	0.20
Diastolic blood pressure, mmHg	68 ± 14	72 ± 11	65 ± 16	0.15
Duration				
Image acquisition, s	573 ± 133	501 ± 92	640 ± 131	** < 0.01**
Manual measurement, s	325 ± 94	296 ± 59	351 ± 110	0.09
AI measurement, s	159 ± 66	110 ± 37	204 ± 50	** < 0.01**
Manual report creation, s	429 ± 128	391 ± 98	464 ± 142	**0.01**
AI report creation, s	71 ± 39	51 ± 29	90 ± 38	** < 0.01**
Image quality fair or poor, *n* (%)	7 (30)	1 (9)	6 (50)	**0.02**
Doppler quality fair or poor, *n* (%)	3 (13)	1 (9)	2 (17)	0.30
Count of modifications	3.8 ± 2.7	2.3 ± 1.9	5.2 ± 2.6	** < 0.01**
Effusion more than mild, *n* (%)	4 (17)	0 (0)	4 (33)	**0.02**
AI report creation				
LV systolic function, *n* (%)	4 (17)	1 (9)	3 (25)	0.17
LV diastolic function, *n* (%)	10 (43)	3 (27)	7 (58)	0.07
LV size, *n* (%)	1 (4)	0 (0)	1 (8)	0.18
LV geometry, *n* (%)	1 (4)	0 (0)	1 (8)	0.18
RV function, *n* (%)	1 (4)	0 (0)	1 (8)	0.18
RV size, *n* (%)	1 (4)	0 (0)	1 (8)	0.18
LA size, *n* (%)	5 (22)	1 (9)	4 (33)	0.09
RA size, *n* (%)	2 (9)	0 (0)	2 (17)	0.09
Aortic stenosis, *n* (%)	1 (4)	0 (0)	1 (8)	0.18
Pulmonary hypertension, *n* (%)	2 (9)	0 (0)	2 (17)	0.09
Clinical considerations, *n* (%)	9 (39)	3 (36)	6 (48)	0.14
Count of diagnosis	1.8 ± 1.9	0.7 ± 0.9	2.8 ± 2.1	** < 0.01**

Data are presented as number of patients or mean ± SD

Bold values signify *p* < 0.05 by *t*-test analysis

LV, left ventricle; RV, right ventricle; LA, left atria; RA, right atria

**Fig. 3:**
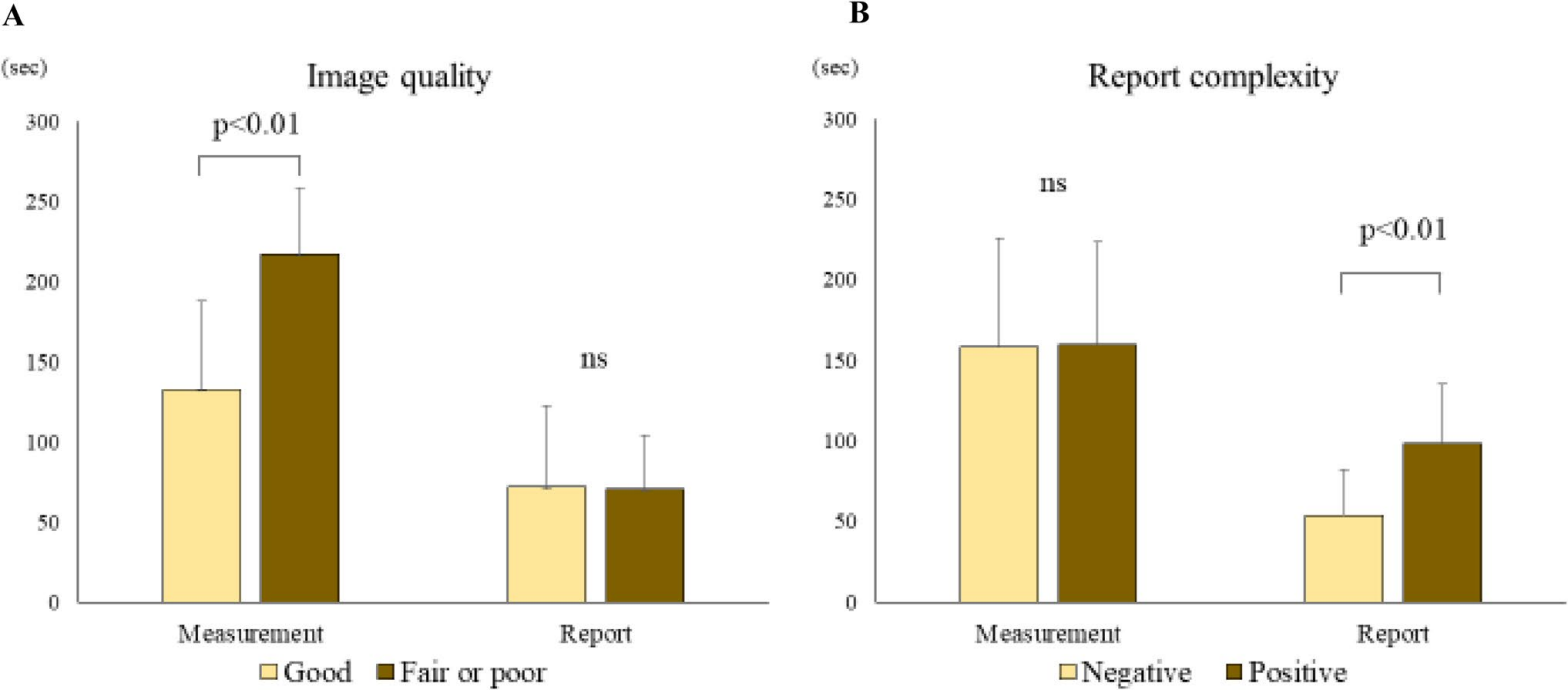
Impact of AI on measurement and report creation time. **A** Difference in image quality; **B** difference with and without findings

The impact of AI on measurement and report creation time was shown in Fig. 3. In cases with fair or poor image quality (*n* = 7), the number of corrections in automated analysis results was higher, and the measurement time significantly increased compared to cases with good image quality (n = 16) (217 ± 51 vs. 133 ± 55 s, *p* < 0.01). However, no significant difference in report creation time was observed based on image quality (72 ± 43 vs. 70 ± 34 s, *p* = 0.25) **(**Fig. 3A**)**.

Regarding the influence of report complexity, no significant difference in measurement time was found between cases with negative (*n* = 14) and cases with positive (*n* = 9) report complexity (158 ± 67 vs. 160 ± 64 s, *p* = 0.18). However, report creation time was significantly longer in cases with positive report complexity compared to cases with negative (99 ± 37 vs. 54 ± 29 s, *p* < 0.01), as it took longer for the human to confirm the findings presented by AI **(**Fig. 3B**)**.

## Discussion

This study conducted a comparative analysis between manual and AI methods in echocardiography, involving 23 consecutive patients. The fully automated AI software exhibited significant potential, reducing echocardiographic analysis time by 70% without compromising accuracy. Patients with faster AI measurements showed a higher frequency of good image quality and a lower number of diagnoses. In cases with fair/poor image quality, more corrections were required, leading to an increase in measurement time. The importance of precise image acquisition by humans was evident, as the obtained measurements directly influenced the report creation process. Overall, the implementation of AI has demonstrated the potential for reducing examination time in the field of echocardiography, thereby making a substantial contribution to enhancing examination efficiency.

### A comprehensive and efficient tool for time savings

In this study, the average time for manual acquisition of routine images was approximately 5–6 min. However, performing measurements for all relevant cardiac parameters post-image acquisition can be time-intensive, often taking 15 min or more, depending on case complexity and the measurer's experience. This can impose a significant burden on examiners. With the Us2.ai cloud-based analysis tool, the measurement time is reduced to less than 1 min for image upload. While there are several reports on time reduction using AI for faster examinations through a semi-automatic approach [17, 18], there are no studies on the extent of time reduction achieved by fully automated software compared to expert manual report creation. Additionally, AI demonstrates nearly 100% recognition and measurement capabilities for the majority of parameters. There were significant ICC observed between AI and expert human measurements, indicating a high level of agreement. AI performs the measurement with extremely good reproducibility and accuracy, as suggested by the findings.

### The applicability of AI in echocardiography

While AI demonstrates high efficiency and accuracy in the majority of cases, it is essential to acknowledge its limitations in certain specific situations or patient characteristic. Particularly, when dealing with fair or poor image quality, the automated analysis necessitated more adjustments to its initial measurements compared to cases with good image quality. Consequently, the time required for automated measurements significantly increased for cases with fair or poor image quality in contrast to those with good image quality. This underscores that the precision of automated measurements could be affected by image quality, emphasizing the need for additional refinements when images are less optimal. A notable aspect of our study was ensuring measurement accuracy, particularly in Doppler imaging. We found that Doppler images rated as 'Fair' for envelope clarity typically matched expert assessments, lessening the need for re-analysis. However, when B-mode images are unclear during Doppler measurements, the automated analysis software may sometimes select incorrect parameters, highlighting the importance of image clarity in both Doppler and B-mode echocardiography for reliable evaluations. Another important finding from the results is that for ultrasound AI tools, image quality plays a crucial role in obtaining reliable automatic measurements [13, 19]. This presents a challenge as ultrasound outcomes are generally influenced by operator skill. While accurate AI tools can be advantageous for less experienced users, the fundamental prerequisite for accurate automatic measurements remains good image quality. This could pose challenges for less experienced examiners. If AI tools exclusively perform well in the hands of experts, their overall utility might come into question. Hence, users should strive to capture optimal images to ensure precise measurements. Nevertheless, despite best efforts, instances of suboptimal image quality may arise. In such scenarios, it is recommended not to solely depend on AI-generated measurements but to adopt a collaborative approach with AI in image evaluation. By working in tandem with AI, more favorable outcomes can be achieved, guaranteeing accurate image assessments for each patient.

### Implications for clinical practice

AI systems consistently produce standardized results, contrasting with the potential variability in outcomes from human echocardiogram technicians due to differences in experience and skills. Additionally, human attempts to expedite the process may introduce measurement errors or mistakes. Therefore, the use of AI leads to improved consistency in test outcomes and reduces the risk of misdiagnosis. Additionally, AI utilization in echocardiograms leads to automatic and rapid result analysis, significantly speeding up report generation compared to traditional methods. As a result, AI adoption enhances result consistency and mitigates the risk of misdiagnosis. Furthermore, integrating AI into echocardiograms automates and expedites result analysis, considerably expediting report generation compared to conventional methods. This time-saving benefit for healthcare professionals allows them to allocate more attention to critical responsibilities like patient examinations and care. Notably, this advancement also positively impacts patients. AI-enabled rapid echocardiogram result delivery shortens waiting times and alleviates anxiety. This fosters a smoother and less stressful medical encounter, ultimately enhancing the overall patient experience. In summary, AI implementation offers multifaceted patient advantages, providing swifter and more dependable results while bolstering healthcare service efficiency and quality.

### Limitations

There are several limitations to this study. First, the study was conducted at a single center, which might restrict the broader applicability of the results. Additionally, the study included only 23 consecutive individuals, resulting in a small sample size that limits the generalizability of the findings to outside populations. Furthermore, the study was carried out by a specific echocardiogram technician, and the results were not compared to those obtained by other examiners, which prevents the assessment of inter-examiner variability or interference.

Moreover, while the study included patients with arrhythmias and poor image quality, it did not consider other diseases or specific clinical situations, potentially limiting the conclusions regarding the applicability of the findings to specific diseases. Another significant limitation is related to the measurement and interpretation time. In our study, the process of importing data into the analysis software and anonymizing it was manually performed, which was time-consuming. However, it is important to note that this issue has been resolved in commercial devices. Additionally, we integrated image interpretation into the measurement process, which hindered our ability to independently assess the interpretation time, particularly in the context of AI methods. Consequently, this approach restricted our capacity to clearly evaluate how interpretation time influences the overall efficiency of measurement and reporting. We identify this as an important focus for future research.

## Conclusions

The fully automated AI software showcases substantial potential for decreasing echocardiographic analysis time while upholding accuracy. This potential offers significant benefits to clinical workflow and efficiency, positively impacting patients and healthcare providers alike. In summary, AI's capacity to expedite and refine echocardiographic interpretation presents a noteworthy stride in medical diagnostics, ultimately resulting in enhanced patient care.

### Supplementary Information

Below is the link to the electronic supplementary material.

Supplementary file1 (DOCX 63 kb)

## Data Availability

The de-identified participant data will not be shared.

## References

[1] Papolos A, Narula J, Bavishi C, et al. Hospital use of echocardiography: insights from the nationwide inpatient sample. J Am Coll Cardiol. 2016;67:502–11. 26846948 10.1016/j.jacc.2015.10.090

[2] Nagueh SF, Abraham TP, Aurigemma GP, et al. Interobserver variability in applying American Society of Echocardiography/European Association of Cardiovascular Imaging 2016 guidelines for estimation of left ventricular filling pressure. Circ Cardiovasc Imaging. 2019;12: e008122. 30632389 10.1161/CIRCIMAGING.118.008122

[3] Bahrami HSZ, Pedersen FHG, Myhr KA, et al. Feasibility, repeatability, and reproducibility of contemporary diastolic parameters and classification. Int J Cardiovasc Imaging. 2021;37:931–44. 33394217 10.1007/s10554-020-02069-z

[4] Oren O, Gersh BJ, Bhatt DL. Artificial intelligence in medical imaging: switching from radiographic pathological data to clinically meaningful endpoints. Lancet Digit Health. 2020;2:e486–8. 33328116 10.1016/S2589-7500(20)30160-6

[5] Vaidyanathan A, Guiot J, Zerka F. An externally validated fully automated deep learning algorithm to classify COVID-19 and other pneumonias on chest computed tomography. ERJ Open Res. 2022. 10.1183/23120541.00579-2021. 35509437 10.1183/23120541.00579-2021 PMC8958945

[6] Kusunose K, Haga A, Inoue M, et al. Clinically feasible and accurate view classification of echocardiographic images using deep learning. Biomolecules. 2020;10:665. 32344829 10.3390/biom10050665 PMC7277840

[7] Kusunose K, Zheng R, Yamada H, et al. How to standardize the measurement of left ventricular ejection fraction. J Med Ultrason. 2001;2022(49):35–43. 10.1007/s10396-021-01116-z PMC8318061 34322777

[8] Zhang J, Deo RC. Response by Zhang and Deo to Letter regarding article, “Fully automated echocardiogram interpretation in clinical practice: feasibility and diagnostic accuracy.” Circulation. 2019;139:1648–9. 30908098 10.1161/CIRCULATIONAHA.119.039291

[9] Ouyang D, He B, Ghorbani A, et al. Video-based AI for beat-to-beat assessment of cardiac function. Nature. 2020;580:252–6. 32269341 10.1038/s41586-020-2145-8 PMC8979576

[10] Papadopoulou SL, Sachpekidis V, Kantartzi V, et al. Clinical validation of an artificial intelligence-assisted algorithm for automated quantification of left ventricular ejection fraction in real time by a novel handheld ultrasound device. Eur Heart J Digit Health. 2022;3:29–37. 36713988 10.1093/ehjdh/ztac001 PMC9707920

[11] Tromp J, Bauer D, Claggett BL, et al. A formal validation of a deep learning-based automated workflow for the interpretation of the echocardiogram. Nat Commun. 2022;13:6776. 36351912 10.1038/s41467-022-34245-1 PMC9646849

[12] Tromp J, Seekings PJ, Hung CL, et al. Automated interpretation of systolic and diastolic function on the echocardiogram: a multicohort study. Lancet Digit Health. 2022;4(1):e46–54. 34863649 10.1016/S2589-7500(21)00235-1

[13] Schuuring MJ, Isgum I, Cosyns B, et al. Routine echocardiography and artificial intelligence solutions. Front Cardiovasc Med. 2021;8: 648877. 33708808 10.3389/fcvm.2021.648877 PMC7940184

[14] Mitchell C, Rahko PS, Blauwet LA, et al. Guidelines for performing a comprehensive transthoracic echocardiographic examination in adults: recommendations from the American Society of Echocardiography. J Am Soc Echocardiogr. 2019;32:1–64. 30282592 10.1016/j.echo.2018.06.004

[15] https://us2.ai/wp-content/uploads/2022/01/FDA-clearance-release-2.0.pdf

[16] Galderisi M, Cosyns B, Edvardsen T, et al. Standardization of adult transthoracic echocardiography reporting in agreement with recent chamber quantification, diastolic function, and heart valve disease recommendations: an expert consensus document of the European Association of Cardiovascular Imaging. Eur Heart J Cardiovasc Imaging. 2017;18:1301–10. 29045589 10.1093/ehjci/jex244

[17] Cecilio-Fernandes D, Cnossen F, Coster J, et al. The effects of expert and augmented feedback on learning a complex medical skill. Percept Mot Skills. 2020;127:766–84. 32228137 10.1177/0031512520914680

[18] Gembicki M, Hartge DR, Dracopoulos C, et al. Semiautomatic fetal intelligent navigation echocardiography has the potential to aid cardiac evaluations even in less experienced hands. J Ultrasound Med. 2020;39:301–9. 31411353 10.1002/jum.15105

[19] Gohar E, Herling A, Mazuz M, et al. Artificial intelligence (AI) versus POCUS expert: a validation study of three automatic AI-based, real-time, hemodynamic echocardiographic assessment tools. J Clin Med. 2023;12:1352. 36835888 10.3390/jcm12041352 PMC9959768

