# Supplementary material for: Reducing echocardiographic examination time through routine use of fully automated software: a comparative study of measurement and report creation time
Source: J Echocardiogr. 2024 Feb 3;22(3):162–70. doi: 10.1007/s12574-023-00636-6 (PMC11343801; doi:10.1007/s12574-023-00636-6)
Supplement: Supplementary file 1 — Supplementary file1 (DOCX 63 kb) [file 12574_2023_636_MOESM1_ESM.docx]

**Reducing Echocardiographic Examination Time through Routine Use of Fully Automated Software: A Comparative Study of Measurement and Report Creation Time**

**Supplemental table 1:** Measurement details

| No | Image quality | Doppler quality | Measurement  (sec) | | Count of modifications | Modified Echocardiographic Indicator |
| --- | --- | --- | --- | --- | --- | --- |
|  |  |  | Manual | AI |  |  |
| 1 | Good | Good | 308 | **57** | 0 | - |
| 2 | Good | Good | 291 | **88** | 2 | Confusion between LVOT Vmax and RVOT Vmax |
| 3 | Good | Good | 335 | **119** | 2 | LVDd, LVDs |
| 4 | Good | Good | 263 | **49** | 0 | - |
| 5 | Good | Good | 303 | **145** | 2 | PWth, LVESV (2ch) |
| 6 | Fair | Good | 258 | **245** | 8 | LVDd, LVDs, PWth, IVSd, LVEDV MOD biplane, LVESV MOD biplane |
| 7 | Good | Good | 326 | **139** | 2 | LVEDV (4ch), LVESV (4ch) |
| 8 | Good | Good | 260 | **255** | 7 | LVDd, PWth, IVSd, LVEDV (4ch), LVESV (4ch), s’ TAM, TAPSE |
| 9 | Good | Good | 199 | **139** | 6 | Confusion between TAM and Sep-TDI |
| 10 | Good | Good | 229 | **132** | 5 | s’ septal, e’ septal, a’ septal, LVEDV (2ch), LVESV (2ch), |
| 11 | Fair | Fair | 300 | **233** | 5 | LVIDd, LVEDV MOD biplane, LVESV MOD biplane |
| 12 | Good | Good | 258 | **150** | 2 | LVdDs, LVEDV (4ch) |
| 13 | Poor | Fair | **225** | 238 | 11 | LVOT Vmax, LVEDV MOD biplane, LVESV MOD biplane  Confusion between TAM and Sep-TDI |
| 14 | Good | Good | 431 | **209** | 4 | LVIDd, LVIDs, PWth, IVSd |
| 15 | Good | Good | 445 | **81** | 2 | PWth, IVSth |
| 16 | Good | Good | 444 | **199** | 5 | LVEDV MOD biplane, s’ lateral, e’ lateral, a’ lateral |
| 17 | Fair | Good | 378 | **292** | 5 | LVIDd, LVEDV MOD biplane, LVESV MOD biplane |
| 18 | Good | Good | 265 | **85** | 1 | DecT |
| 19 | Fair | Good | 267 | **156** | 6 | LVIDd, LVIDs, PWth, IVSd, LVEDV (2ch), LVESV (2ch) |
| 20 | Fair | Good | 336 | **132** | 3 | DecT, LVEDV (4ch), LVESV (4ch) |
| 21 | Good | Good | 349 | **109** | 0 | - |
| 22 | Good | Good | 632 | **174** | 3 | LVPWth, IVSd, AoV Vmax |
| 23 | Fair | Fair | 375 | **226** | 6 | LVPWth, IVSd, LVEDV MOD biplane, LVESV MOD biplane |

Abbreviations: IVSd, interventricular septal thickness in diastole; LVIDd, left ventricular internal diameter in diastole; LVIDs, left ventricular internal diameter in systole; LVPWd, left ventricular posterior wall thickness in diastole; LVEDV MOD biplane, left ventricular end diastolic volume by the modified Simpson's biplane method; LVESV MOD biplane, left ventricular end systolic volume by the modified Simpson's biplane method; LVOT Vmax, left ventricular outflow tract peak velocity; RVOT V max, right ventricular outflow tract peak velocity; AoV Vmax, aortic valve peak velocity; DecT, deceleration time; e’ lateral, early diastolic tissue Doppler velocity at the lateral mitral annulus; e’ septal, early diastolic tissue Doppler velocity at the septal mitral annulus; a’ lateral, late diastolic tissue Doppler velocity at the lateral mitral annulus lateral; a’ septal, late diastolic tissue Doppler velocity at the septal mitral annulus; s’ lateral, systolic tissue Doppler velocity at the lateral mitral annulus; s’ septal, systolic tissue Doppler velocity at the septal mitral annulus; TR V max, tricuspid regurgitant peak velocity; TAPSE, tricuspid annular plane systolic excursion; S’ TAM, systolic tissue Doppler velocity at the tricuspid annulus.

**Supplemental table 2:** Report details

| No | Purpose | Report  (sec) | | Report complexity | Positive findings |
| --- | --- | --- | --- | --- | --- |
|  |  | Manual | AI |  |  |
| 1 | Screening | 463 | **39** | 1 | LV diastolic dysfunction, Clinical considerations |
| 2 | Screening | 320 | **109** | 0 | LV diastolic dysfunction |
| 3 | Screening | 363 | **63** | 0 | - |
| 4 | Screening | 358 | **19** | 0 | - |
| 5 | Screening | 312 | **19** | 0 | - |
| 6 | Screening | 343 | **48** | 0 | - |
| 7 | Screening | 474 | **51** | 0 | - |
| 8 | Screening | 266 | **39** | 0 | LV diastolic dysfunction |
| 9 | Screening | 299 | **69** | 0 | - |
| 10 | Screening | 385 | **85** | 0 | Effusion |
| 11 | Screening | 288 | **44** | 0 | LV diastolic dysfunction |
| 12 | Screening | 347 | **20** | 0 | LV diastolic dysfunction |
| 13 | Screening | 316 | **36** | 0 | Effusion |
| 14 | ICM | 685 | **126** | 1 | LV diastolic dysfunction, LV systolic dysfunction, Clinical considerations, Effusion |
| 15 | ICM | 650 | **96** | 1 | LV systolic dysfunction, Clinical considerations |
| 16 | ICM | 602 | **153** | 1 | LV diastolic dysfunction, LV systolic dysfunction, LV size, RA size, Pulmonary hypertension, Clinical considerations |
| 17 | ICM | 426 | **125** | 1 | LV diastolic dysfunction, LV systolic dysfunction, LV size, LA size, Clinical considerations |
| 18 | Arrhythmia | 336 | **36** | 0 | - |
| 19 | Arrhythmia | 383 | **42** | 1 | LA size, Clinical considerations |
| 20 | Severe PH | 565 | **89** | 1 | RV function, RVsize, RA size, Pulmonary hypertension, Effusion, Clinical considerations |
| 21 | HCM | 668 | **128** | 1 | LV Geometry, LA size, Clinical considerations, |
| 22 | Severe AS | 556 | **95** | 1 | LV Diastolic dysfunction, Aortic stenosis, Clinical considerations, LA size |
| 23 | HFpEF | 469 | **109** | 0 | LV diastolic dysfunction |

Abbreviations: ICM, ischemic cardiomyopathy; PH pulmonary hypertension; HCM, hypertrophic cardiomyopathy; AS, aortic stenosis; HFpEF, heart failure with preserved ejection fraction.
